# Left flank pain during pregnancy with an unpredictable etiology: think of nonhemorrhagic adrenal infarction

**DOI:** 10.2144/fsoa-2021-0022

**Published:** 2021-05-28

**Authors:** Myriam Jerbaka, Tracy Slaiby, Zahraa Farhat, Yara Diab, Nawal Toufayli, Khaled Rida, Taghreed Diab

**Affiliations:** 1 Department of Gynecology & Obstetrics, Saint Joseph University, Lebanese University, Bahman University Hospital, Beirut, Lebanon; 2 Department of Gynecology & Obstetrics, Lebanese American University, Rizk University, Beirut, Lebanon; 3 Department of General Surgery, Bahman University Hospital, Beirut, Lebanon; 4 Department of Gynecology & Obstetrics, Bahman university hospital, Beirut Lebanon

**Keywords:** adrenal infarction, case report, flank pain, oligohydramnios, pregnancy, preterm birth

## Abstract

Abdominal pain is the most presenting complaint during pregnancy with multiple etiologies. The diagnosis could be unpredictable. We present a case of 36-year-old pregnant woman *gravida* 10 *para* 7 *abortus* 2 at 36 + 5 weeks of gestation presenting twice for an increasing left abdominal pain, not relieved despite analgesics. She was delivered for severe oligohydramnios. After delivery, she was found to have a left adrenal infarction on computed tomography scan. She was found to have two mutations of the gene *MTHFR 677CC*. Our presented case should remind physicians to consider the presence of thromboembolic state during pregnancy. The diagnosis of adrenal infarction should be among the differentials of an ambiguous flank pain that is resilient to medical therapy. Diagnosis in a pregnant patient can be easily confirmed with MRI, after which anticoagulation should be started and the workup for hypercoagulable state investigated.

## Lay abstract

A 36-year-old pregnant woman during her tenth pregnancy, known to have seven previous normal vaginal deliveries, presented at 36 weeks and 5 days of her gestational age with increasing left flank pain. She was found to have a left adrenal gland infarction on computed tomography scan after delivery. She has no previous medical history. There was no evidence of adrenal insufficiency during pregnancy. Evaluation for hypercoagulable state showed two mutations in *MTHFR 677CC*. The patient was started on anticoagulation therapy to prevent right adrenal thrombosis. She received treatment by anticoagulation for 1 week and was discharged on prophylactic dose for 6 months.

Abdominal pain is the most common chief complaint during pregnancy. It is a routine priority to rule out uterine contractions by nonstress test in pregnant women complaining of flank pain, low back pain or pelvic pain. However, if labor pain is excluded, what else can be added to the differentials of flank pain during pregnancy and what are the best testing modalities?

During pregnancy, identifying the cause of pain seems to be more challenging due to the physiologic changes that can affect some laboratory results and due to some restrictions in testing and even interventions. Eventually, the goal is to exclude serious or even life-threatening etiologies by reaching a prompt diagnosis and treatment, otherwise unnecessary delay will risk increasing maternal, fetal or newborn morbidity and mortality.

In this article, we will present a very rare cause of pain with an atypical presentation during the late weeks of pregnancy. The diagnosis was only made in the postpartum period when the symptom did not improve but even worsened. The challenging diagnosis was a nonhemorrhagic left adrenal infarction.

## Case presentation

A 36-year-old woman *gravida* 10 *para* 7 *abortus* 2 at 36 + 5 weeks of gestational age, presented for two consecutive days complaining of an increasing new onset of acute left upper quadrant pain. The pain was constant with intermittent worsening in her left upper quadrant and radiating to her left flank. During this gestation, she reported a previous presentation for abdominal pain with nausea lasting for 1 week. Those symptoms were associated with gastritis because no other cause was found.

The patient initially presented to the hospital for an increasing pain that was believed to be due to fetal movements or uterine contractions. She was given antispasmodics. However, the pain intensified which was the reason for her to seek medical advice again. She did not complain of any nausea or vomiting, nor decrease in appetite but she had 2 days of constipation. The patient’s medical history was unremarkable. Her obstetrical history included two spontaneous pregnancy losses that needed dilation and curettage at first trimester. She also had previous seven uncomplicated vaginal deliveries.

On presentation to the hospital, her blood pressure was 110/70 mmHg, her pulse was 84 beats per min, temperature was 36.9°C, saturation of O_2_ was 100%, respiratory rate was 20.

On physical examination, the patient appeared uncomfortable and agitated.

Her abdomen was soft; the uterus was gravid with no fundal tenderness. Bowel sounds were present and normal. There was moderate tenderness on the left upper quadrant under the rib cage with mild rebound. She also had costovertebral angle tenderness on the left side. The patient was admitted to the delivery suite and initially was started on intravenous fluid hydration, proton pump inhibitor and analgesia. This provided minimal relief to the patient. Nonstress test monitoring was done at this time also showing reassuring reactivity of the fetal heart rate with no contractions.

Several differential diagnoses are to be noted: renal colic, mesenteric infarction, splenic vein thrombosis, pregnancy associated pain, pancreatitis and spleen associated pain.

A complete set of laboratory tests were done and were unremarkable except for microcytic anemia hemoglobin was 8.9 g/dl and hematocrit was 29%. She had normal liver enzymes, pancreatic enzymes and creatinine.

An abdominopelvic ultrasound was performed which showed oligohydramnios and sludge in the gallbladder. The appendix was not visualized, no gallstones, no biliary ducts dilation, no hydronephrosis. Doppler of the splenic vessels was not done, although splenic vein thrombosis was one of the differential diagnoses.

At this time induction of labor was decided due to oligohydramnios. The patient delivered a live born boy APGAR score 9 and 10 at 1 and 5 min weighing 3040 grams.

In postpartum period, the pain persisted and; therefore, surgical consultation recommended CT scan of the abdomen with contrast to rule out pancreatitis and splenic vein thrombosis. Subsequently the CT scan revealed left adrenal gland with decreased enhancement and adjacent inflammatory changes suggestive of an infarct. Right adrenal gland was normal. Normal appearing appendix, no anomalous vasculature was seen and MRI study was unlikely to add any more information; therefore, was not done.

Cortisol level and adrenocoticotropic hormone level were obtained to assess for adrenal insufficiency. The hemato-oncologist was consulted to workup the thrombophilia disorders that included testing for factor V Leiden, prothrombin mutation and MTHFR as well as antiphospholipid panel, protein C and S, antithrombin 3, factor II, factor XIII, plasminogen activator inhibitor-1 mutation and annexin A5 antibodies.

There was no adrenal insufficiency and thrombophilia workup showed MTHFR C677T homozygous mutation and HPA1 1a/1b heterozygous mutation. The results of workup of coagulopathies, adrenal insufficiency as well as thrombophilia disorders are shown in (Tables 1 & 2), respectively.

**Table 1.** Results of workup for adrenal insufficiency and coagulopathy.

Name	Result	Unit
Fibrinogen	319	mg/dl
CORT II	783.4	nmol/l
Anti-B 2 glycoprotein I IgA, IgG, IgM	8.06	Ru/ml
Ac anticardiolipine IgA, IgG, IgM	<2	Ru/ml
Antinuclear antibody	negative	
ACTH	23	pg/ml
Protein C	80	%
Protein S	55	%
Antithrombin III	85	%

ACTH: Adrenocorticotropic hormone; CORT II: Cortisol level.

**Table 2.** Results of thrombophilia workup.

Gene	Codon	Result
Factor V	G1691A (Leiden)	No mutation detected
Factor V	H1299R (R2)	No mutation detected
Factor II prothrombin	G20210A	No mutation detected
Factor XIII	V34L	No mutation detected
B-fibrinogen	-455G >A	No mutation detected
PAI-1	4G/5G	5G/5G
Platelet glycoprotein IIIa GPIIIa (HPA1)	a/b	1b/1a
MTHFR	C677T	Two mutations
MTHFR	A1298C	No mutation
ACE	Deletions/insertion	D/1
Apo B	R3500Q	No mutation
Apo E	Codon 112:TGC (Cys)	+
Apo E	Codon 112: CGC (Arg)	−
Apo E	Codon 158: TGC (Cys)	−
Apo E	Codon 158: CGC (Arg)	+

ACE: Angiotensin-converting enzyme; Apo: Apolipoproteins; MTHFR: Methylene tetrahydrofolate reductase; PAI: Plasminogen activator inhibitor-1.

Anticoagulation with low molecular weight heparin (LMWH) 40 mg subcutaneously (sc.) once daily was initiated to reduce the risk of another infarct in the contralateral adrenal gland. After 2 days, she was started on therapeutic dose LMWH 60 mg sc. for 1 week.

**Figure 1.**
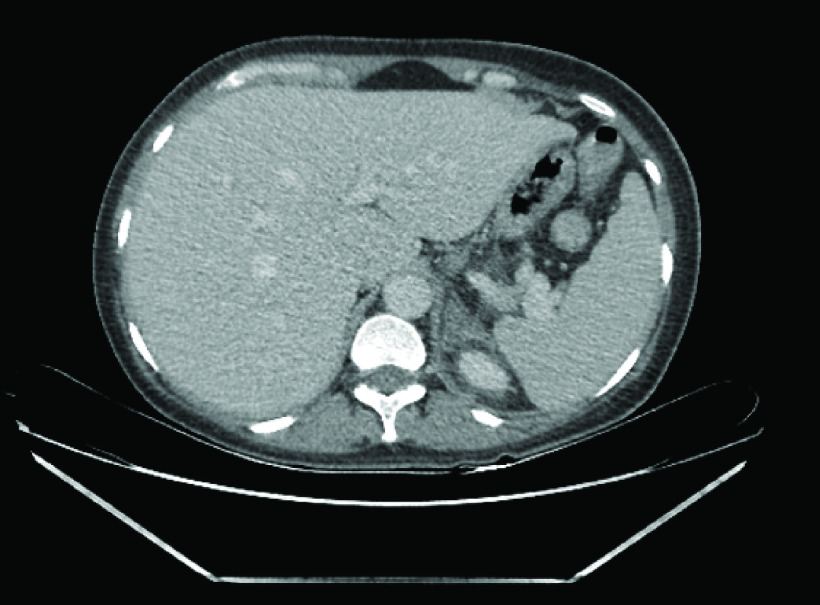
Computed tomography scan postpartum showing nonhemorragic adrenal infarction on the left side.

**Figure 2.**
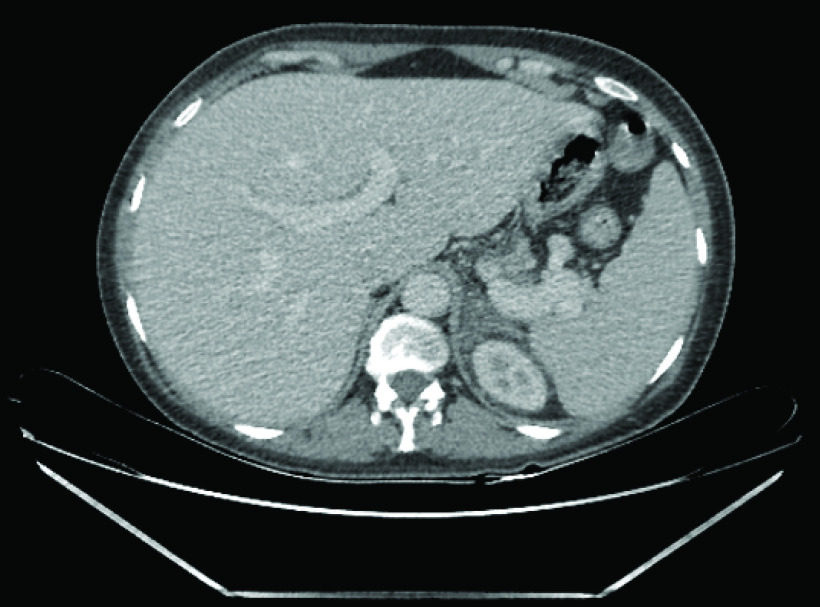
Computed tomography scan showing the left kidney and the left infarcted edematous adrenal gland in the immediate postpartum period.

After 4 days the patient was discharged home for outpatient postpartum care on prophylactic LMWH for 6 months. (Figures 1 & 2).

**Figure 3.**
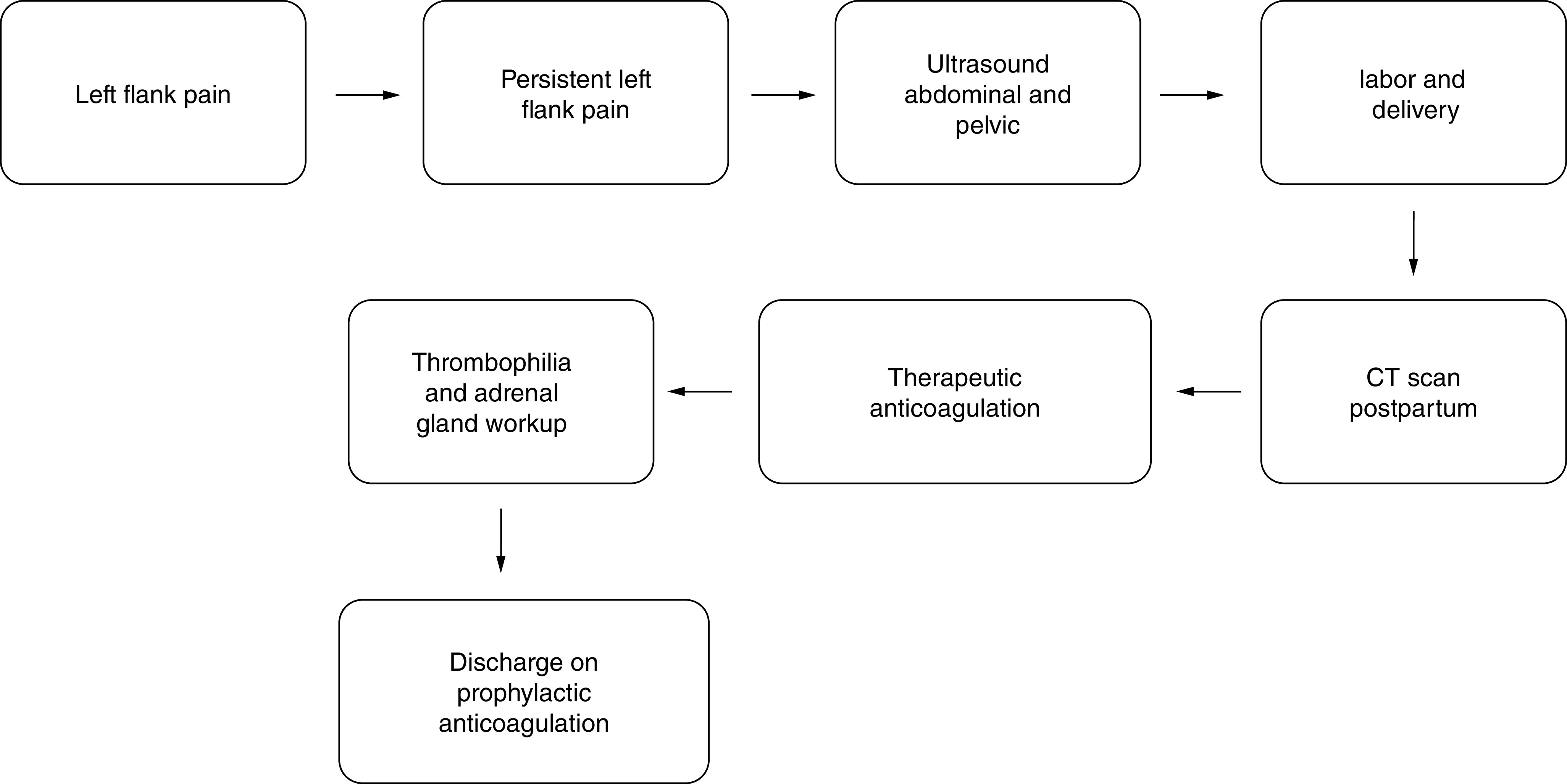
Timeline of patient. CT: Computed tomography.

During the 6 months follow-up, the patient did not have any complaints and was clinically and hemodynamically stable. She had to continue iron supplements because of her iron deficiency anemia. A timeline can be seen in Figure 3.

## Discussion

Nonhemorrhagic adrenal infarction (NHAI) is a very rare diagnosis with only a few reported cases during pregnancy [1–11]. We have found around nine cases in the literature describing unilateral NHAI during pregnancy [1–3,5–7,9,12].

Adrenal infarction can present with abdominal pain, nausea and vomiting as well as electrolytes disturbances. The adrenal glands are endocrine glands located in the retroperitoneum just above the kidneys and are responsible for secreting hormones in response to stress and releasing androgens.

The adrenal gland has rich blood supply via three main arteries: the superior adrenal artery arising from the inferior phrenic artery, the middle artery from the abdominal aorta and the inferior adrenal from the renal artery. However, the venous blood draining the glands by the right and left adrenal veins only. The right adrenal vein drains into the inferior vena cava whereas the left adrenal vein drains into the left renal vein.

Therefore, altered venous drainage with increased arterial blood flow can lead to adrenal blood stasis and infarction. The localization of the thrombi in adrenal vein might result in local stasis of blood leading to edema and necrosis of the gland in hypercoagulable states.

The case we are presenting in this article highlights two important issues. First, despite that adrenal infarction is a very rare diagnosis, it should be taken into consideration as one of the differential diagnoses when a pregnant woman presents for acute severe abdominal pain. Second, once a diagnosis of NHAI is established clinically and radiologically, we should search for a thrombophilic and hypercoagulable state.

A small number of cases of NHAI has been reported. Nine cases of unilateral NHAI during pregnancy have been published in the literature. All cases have similar presentation: severe unremitting acute abdominal pain, resisting usual analgesia with other clinical signs that are mild and not specific. Ultrasound imaging is usually not conclusive and diagnosis was definitive by CT scan or MRI. Most of these patients received anticoagulation as therapy and prophylaxis from the contralateral gland thrombosis. As of now, the role of therapeutic dose of anticoagulation has not been proven to be efficient and; therefore, prophylactic dose is usually given in this situation to prevent any further events.

In two cases of Green *et al.* and Sormunen-Harju *et al.* [1,5], both patients had significant results of heterozygous MTHFR C677T polymorphism gene mutation whereas another one [13] was associated with antiphospholipid syndrome. Another case was associated with factor VIII [2].

The interesting part is that all nine cases involved the right adrenal gland while in our case, the left adrenal gland was the infarcted one. We also know that the main involvement of the right adrenal vein could be explained by its anatomical location with respect to that of the vena cava taking into account that the return of venous blood can be impaired by dextroverted gravid uterus leading to adrenal blood stasis. In other rare cases, when the ischemia is bilateral, adrenal insufficiency can occur.

Literature supports evaluation with CT or MRI to make a diagnosis of suspected adrenal infarction or hemorrhage [9–11]. The gold standard for diagnosing NHAI is contrast-CT that displays diffuse adrenal gland enlargement, hypo-enhancement and adjacent fat stranding and fluid in the retroperitoneum. On the other hand, during pregnancy, the exposure of the fetus to radiation needs to be minimized. To the best of our knowledge, a noncontrast MRI should be performed if the pain is occurring in the preterm period even though MRI could be inconclusive and a CT scan could be needed. In our case, the patient was 36 + 5 weeks of gestation which made her close to term and she needed induction of labor for oligohydramnios; therefore, a contrast-CT scan was postponed till the postpartum period.

Once the diagnosis is made, prophylactic anticoagulation should be initiated as by the recommendations to prevent contralateral adrenal gland infarction.

The risk of anticoagulation should be considered especially in the presence of hemorrhage or if the infarct occurred before delivery; we should take into consideration the potential complications with regional anesthesia or bleeding related to vaginal or cesarean delivery.

The mechanism of spontaneous adrenal infarction is different from that of hemorrhage due to trauma, underlying tumor or systemic infection as seen in Waterhouse–Friderichsen syndrome associated with meningococcemia.

The features described on MRI (retroperitoneal edema centered on the adrenal, elongation of the infarcted adrenal and increased T2 signal intensity of the infarcted adrenal compared with the contralateral one) also correlate with previous literature describing the CT appearance of adrenal infarction [4].

In our patient, the presumed diagnosis of nonhemorrhagic adrenal infarction was supported by clinical presentation, laterality of symptoms matching the abnormal adrenal appearance on CT scan and absence of another pathological entity explaining the presentation.

Therefore, the diagnosis of adrenal infarction should be suspected clinically in pregnant patients with unilateral abdominal or flank pain which is often severe, associated with nausea and vomiting like in our patient. Upon physical examination, presence of tenderness localized to the symptomatic area is noted but without peritoneal signs although sometimes it may be unremarkable.

Our patient did not have adrenal insufficiency signs or symptoms. Therefore, a diagnosis of adrenal infarct during pregnancy is challenging. To diagnose an adrenal infarction, a great degree of clinical suspicion is required.

During normal pregnancy, maternal adrenal glands will undergo changes. The cortisol physiology results in both total and free cortisol concentrations rising throughout the gestation. The increased estrogen in pregnancy stimulates corticosteroid-binding globulin level and; therefore, decreases free cortisol levels. As pregnancy progresses, an increase in pituitary adrenocorticotropic hormone production and an increase in free cortisol levels will result in an equal and striking rise into the third trimester.

Treatment of adrenal infarction in pregnancy is based on narcotics for pain control, hematologic evaluation and anticoagulation therapy; however, some physicians have advocated conservative management without anticoagulation. There are insufficient long-term outcomes or understandings of the pathophysiology of the necrosis; therefore, it is difficult to favor either management approach.

The foremost limitation of our case is that the etiology of infarction was not very well established because the only finding was homozygous C677T mutation of the MTHFR gene. On the other hand, optimal treatment of patients with NHAI with or without anticoagulation remains to be determined and should be decided on patient-by-patient basis by means of hematologic evaluation [14].

## Conclusion

During pregnancy, one of the most common chief complaints are abdominal and pelvic pain. Multiple differential diagnoses could be related to these complaints. One of the very rare ones is adrenal infarction. We report a case of NHAI in a multigravida patient during the beginning of her term and the approach for the diagnosis and the management; although, it is still not very well known what the best management is for long-term outcomes and what the real etiology of this disease is. Further cases should be recognized and more investigations should be done in order not to miss diagnosis as it might be life-threatening.

The case has been reported with the CARE guidelines for case reporting.

## Future perspective

The diagnosis of adrenal infarction should be considered in pregnant patients presenting for acute unilateral abdominal or flank pain. This pain is often severe and accompanied by other symptoms such as nausea and vomiting and even labor pain. Pregnancy alone is a risk factor for thromboembolism. The diagnosis should also be under more suspicion in patients with known risk factors for thrombosis. If not known, more investigations should be done for the patient for coagulopathies and thrombophilias and adrenal insufficiency should be ruled out. An MRI should be the gold standard imaging during pregnancy and should be ordered when the cause of pain is not well established. Treatment with a therapeutic dose of anticoagulation might lead to better outcomes and might heal the infarcted adrenal gland. Meanwhile prophylactic anticoagulation should be given if there is still no evidence of the therapeutic effect of anticoagulation on long-term outcomes and recovery of the infarcted gland.

**Executive summary**

- Abdominal pain during pregnancy has multiple etiologies and it is sometimes difficult to predict the diagnosis.
- Nonhemorrhagic adrenal infarction is one of the very rare diagnosis causing abdominal and flank pain during pregnancy.
- The etiology of nonhemorrhagic adrenal infarction is not very well established. Most of the cases reported suggests that it is a due to coagulopathy. Therefore, a workup of coagulopathy and thrombophilic disorders should be done.
- Treatment, although not very clear, is with anticoagulation to avoid infarct of the contralateral adrenal gland.
